# The effects of normal aging on multiple aspects of financial decision-making

**DOI:** 10.1371/journal.pone.0182620

**Published:** 2017-08-09

**Authors:** Dorien F. Bangma, Anselm B. M. Fuermaier, Lara Tucha, Oliver Tucha, Janneke Koerts

**Affiliations:** Department of Clinical and Developmental Neuropsychology, University of Groningen, Groningen, The Netherlands; Universidad de Granada, SPAIN

## Abstract

**Objectives:**

Financial decision-making (FDM) is crucial for independent living. Due to cognitive decline that accompanies normal aging, older adults might have difficulties in some aspects of FDM. However, an improved knowledge, personal experience and affective decision-making, which are also related to normal aging, may lead to a stable or even improved age-related performance in some other aspects of FDM. Therefore, the present explorative study examines the effects of normal aging on multiple aspects of FDM.

**Methods:**

One-hundred and eighty participants (range 18–87 years) were assessed with eight FDM tests and several standard neuropsychological tests. Age effects were evaluated using hierarchical multiple regression analyses. The validity of the prediction models was examined by internal validation (i.e. bootstrap resampling procedure) as well as external validation on another, independent, sample of participants (n = 124). Multiple regression and correlation analyses were applied to investigate the mediation effect of standard measures of cognition on the observed effects of age on FDM.

**Results:**

On a relatively basic level of FDM (e.g., paying bills or using FDM styles) no significant effects of aging were found. However more complex FDM, such as making decisions in accordance with specific rules, becomes more difficult with advancing age. Furthermore, an older age was found to be related to a decreased sensitivity for impulsive buying. These results were confirmed by the internal and external validation analyses. Mediation effects of numeracy and planning were found to explain parts of the association between one aspect of FDM (i.e. Competence in decision rules) and age; however, these cognitive domains were not able to completely explain the relation between age and FDM.

**Conclusion:**

Normal aging has a negative influence on a complex aspect of FDM, however, other aspects appear to be unaffected by normal aging or improve.

## Introduction

During adulthood, most people make financial decisions. Some of these decisions are age or life stage dependent, such as buying a house or saving for retirement, while other decisions occur throughout adulthood, such as buying groceries and paying bills. Financial decision-making (FDM) is thus necessary in multiple situations and is crucial for independent living.

FDM requires the integrity of cognition [1] and appears to be vulnerable to cognitive changes and dysfunctioning of especially the prefrontal cortex [2,3]. Consequently, it is not surprising that impairments in FDM are found in patients with different brain pathologies, such as patients with Alzheimer’s disease, Mild Cognitive Impairment and Parkinson’s disease [4–10]. Normal aging is, however, also accompanied by structural and functional changes of the prefrontal cortex [11,12]. These age-related changes might result in a decreased memory, attention and executive functioning [13,14] and consequently in deficits of FDM in older individuals.

Previous studies have shown that deliberative information processing is essential for decision-making. Deliberative information processing can be defined as a rational manner of manipulating information that is needed to come to a decision (e.g. [15]). Deliberative information processing is thought to rely strongly on working memory capacity and related cognitive functions [16]. Indeed, previous research indicates that deliberative information processes are less efficient with advancing age due to an age-related cognitive decline in processing speed, short- and long-term memory, working memory, attention, inhibition and reasoning [13,14,17,18]. As a result, older adults evaluate less information before making a decision [19] and have more difficulties with rather complex decision-making [20,21] than younger individuals. Furthermore, in de context of FDM, also numeracy abilities, independent of age, education level and other cognitive functions, are thought to play an important role [22–25].

Besides deliberative information processes, affective processes were also found to play a role in decision-making (e.g. [26,27]). Affective processing relies on personal experience and prior learning on how to deal with certain decision-making situations [15]. Not only integral affect (i.e. the feelings or emotions towards a problem or decision), but also incidental affect (i.e. the emotional status, unrelated to the problem or decision, while making a decision) are related to affective processing and have an influence on decision-making [15]. It has been suggested that these affective processes remain unaffected or even improve with advancing age [15,28,29]. Furthermore, also personal experience in and knowledge about (financial) decision-making (e.g. the experience with paying bills or buying or selling a property) expand with advancing age [15,30]. Affective processing and personal knowledge and experience might, therefore, compensate for a decreased deliberative cognitive processing in older adults resulting in similar or even better decision-making with advancing age [31,32]. It can thus be concluded that normal aging may have negative effects as well as positive influences on (financial) decision-making.

So far, only a few studies focused on FDM. These studies primarily examined an older (demented) population and investigated relatively basic aspects of FDM, such as the ability to recognize coins, count money and pay bills (e.g. [4–6]). FDM, however, also includes buying or selling property, saving for retirement and taking out health insurances. Furthermore, unfitting behavior in a financial context, such as impulsive buying and gambling, can lead to major consequences for an individual’s financial situation. Therefore, to gain a comprehensive overview of an individual’s FDM skills and abilities it is of utmost importance to not only assesses the simple aspects of FDM but also to study more complex aspects of FDM.

The aim of the present explorative study is to determine the effects of normal aging on multiple aspects of FDM. Therefore, based on previously published procedures, a test battery was developed encompassing eight FDM tests measuring eight aspects of FDM (i.e., financial competence, (financial) decision-making capacity, financial decision styles, ability to apply rules, decisions with implications for the future, impulsive buying tendency, emotional decision-making and intuitive and deliberative decision-making). It is expected that an advancing age results in a decreased performance in aspects of FDM that rely strongly on deliberative information processing (e.g. the ability to apply rules) due to an age-related cognitive decline that accompanies normal aging [13,14,17,18]. However, it is also expected that other aspects of FDM, that rely more on knowledge or affective processing, are not affected by aging (e.g. financial competence) or even improve with advancing age (e.g. impulsive buying or intuitive decisions) due to an age-related increase in knowledge and experience with FDM or an improved affective decision-making in older adults [15,28–30]. Furthermore, since this is an explorative study, it is important to determine whether or not our results are due to statistical errors and can be replicated. Therefore, the validity of the prediction models will be examined by internal validation (i.e. bootstrap resampling procedure) as well as external validation on another, independent, sample of participants. Finally, the association between cognitive functions and the different aspects of FDM and the possible mediating effect of cognition on the relation between age and FDM are investigated.

## Materials and methods

### Participants—Sample 1

One hundred and ninety-one adults participated in this study. Participants were recruited through advertisement in a local newspaper and via social contacts of the researchers (i.e. family members and acquaintances of the researchers and of included participants were informed about the study via word-of-mouth, email or social media). Eleven participants with evidence for psychiatric or neurological conditions were excluded: five participants showed high scores on a depression rating scale (Beck Depression Inventory-II-NL > 19 [33]), two participants obtained low scores on a dementia rating scale (Mini Mental State Examination < 24 [34]) and four participants reported having a neurological or psychiatric condition which might have a negative impact on cognition (e.g. brain tumor and Lyme disease). The remaining 180 participants (sample 1) were on average 49.58 years old (range 18–87 years; Table 1) and obtained on average 15.15 years of education. Slightly more females (57.2%) than males were included in sample 1 (Table 1).

**Table 1:** Demographic characteristics of samples 1 and 2.

	Sample 1	Sample 2	Group differences (p-value)
n	180	124	
Age range	18–87	19–87	
Age M(SD)	49.58 (18.37)	41.28 (17.73)	< .001^a^
Age groups			< .001^b^
< 30	20.6%	37.9%	
30–39	12.2%	14.5%	
40–49	11.1%	5.6%	
50–59	22.2%	27.4%	
60–69	21.7%	7.3%	
> 70	12.2%	7.3%	
Female	57.2%	58.9%	.775^c^
Education M(SD)	15.15 (3.78)	17.04 (3.60)	< .001^a^
In employment	61.1%	67.7%	.237^c^
Income range	25.000–35.000	25.000–35.000	.198^b^

Note. Age and Education in years; Average annual gross income range in Euro.

^a^ one-way ANOVA,

^b^ Mann-Whitney U test or

^c^ Pearson’s chi-square tests.

### Participants—Sample 2

A second sample (n = 124) was included to evaluate the external validity of possible age effects. The recruitment procedure (i.e. via contacts of the researchers, recruited by word-of-mouth, e-mail or social media) as well as the inclusion and exclusion criteria (e.g. no evidence for a psychiatric or neurological condition) were similar to the first sample. None of the participants in sample 1 were also included to sample 2. Both samples were comparable with regard to the age range. However, the average age of the second sample was significantly lower than in the first sample (Table 1). Furthermore, the second sample obtained on average two more years of education than the first sample. The samples did not differ significantly on other demographic characteristics (Table 1).

### Ethics statement

The Ethical Committee Psychology of the University of Groningen, Groningen, The Netherlands, and the Medical Ethical Committee of the University Medical Center Groningen, Groningen, The Netherlands, approved this study. Participants were informed about the content of the research prior to assessment. Furthermore, they were informed that participation was voluntary and that they were able to withdraw at any time. All participants signed a written informed consent prior to assessment.

### Procedure and measurement

The assessment in sample 1 consisted of eight FDM tests and several standard neuropsychological tests. All tests were performed consecutively and in a fixed order with an approximate duration of 3.5 hours (see S1 Procedure for an overview of the sequence of tests). The fixed sequence of tests was chosen to minimize the interference between cognitive tests. One break of at least fifteen minutes was included, however, participants could ask for as many breaks as needed to minimize the effects of fatigue. Questionnaires were completed at home prior to assessment. Each participant was tested individually. The data of sample 2 was collected in the context of another study and in this study only FDM tests were used.

#### Tests measuring financial decision-making

The Financial Competence Assessment Inventory—NL (FCAI-NL) is an in Dutch translated and updated version of the original FCAI task developed by Kershaw and Webber [6,35]. The FCAI-NL provides information about strengths and weaknesses regarding an individual’s ability and knowledge to perform actions that are needed to execute financial transactions on a relatively fundamental level (further described as ‘financial competence’ [35,36]). The original FCAI appeared to be suitable for different age and patient groups [6,35]. The FCAI-NL consists of six different subscales based on the six dimensions of financial competence [6,35]: ‘Financial abilities’ (e.g. the ability to understand the information that is presented on bills), ‘Financial judgment’ (e.g. identifying and understanding items on a bank statement), ‘Financial cognitive functioning’ (e.g. writing a cheque), ‘Financial management’ (e.g. understanding banking protocols), ‘Debt management’ (e.g. awareness of own debts) and ‘Financial support resources’ (e.g. assistance seeking skills). In total, the FCAI-NL contains 38 questions varying between theoretical questions, practical assignments and questions about the financial situation of the participant. For each question, participants receive a score between 0 (no awareness) and 4 (complete understanding) depending on the accuracy of the answer, with the exception of yes/no questions (score of 0 or 1 points, respectively). The total score of the FCAI-NL (maximum score of 134) is based on the sum of the six subscales and gives an indication of an individual’s overall financial competence.

The Financial Decision-Making Interview (FDMI) is a newly developed test used to determine the mental ability or capacity to make a decision related to financial issues (i.e. (financial) decision-making capacity). Decision-making capacity consists of five domains [37,38], i.e. the ability to identify a problem (‘Identification’), to understand information and consider risks and benefits (‘Understanding’), to reason about different options and make a reasoned decision (‘Reasoning’), to appreciate and evaluate the effects of decisions (‘Appreciating’) and to articulate a final choice (‘Communication’). The principle of the FDMI is based on the MacArthur Competence Assessment Tool and on previously published procedures that were used to assess (medical) decision-making [37–42]. In the FDMI, two vignettes with a complex hypothetical financial problem are presented in oral and written form. After the presentation of the vignette, participants are asked to answer questions related to the five domains of decision-making capacity. For each answer, participants receive a score of 0, 1 or 2 based on the degree of completeness. The scores on both vignettes are combined and one overall total score (maximum score of 20) is calculated as well as total scores for each subscale (maximum score of 4).

The Financial Decision Style (FDS) questionnaire is used to evaluate the manners individuals apply in FDM situations (i.e. financial decision styles) [43,44]. The FDS is based on the General Decision-Making Style questionnaire [44] which is a self-report questionnaire with good construct validity [45,46]. The FDS consists of 24 questions and differentiates five decision-making styles [44], i.e. (1) a ‘rational’ style characterized by the evaluation of options (e.g. ‘I make financial decisions in a logical and systematic manner’), (2) an ‘intuitive’ style which allows individuals to rely on feelings or emotions (e.g. ‘When I make financial decisions, I tend to rely on my intuition’), (3) a ‘dependent’ style characterized by the requirement of advice of others (e.g. ‘I rarely make important financial decisions without consulting other people’), (4) an ‘avoidant’ style characterized by avoiding and postponing decisions (e.g. ‘I postpone financial decision-making whenever possible’) and (5) a ‘spontaneous’ style characterized by impulsivity (e.g. ‘I often make financial decisions in the spur of the moment’). For each question, participants are asked to rate on a scale ranging from 1 (strongly disagree) to 5 (strongly agree) to what extent each situation applies to them. A total score is calculated for each financial decision style.

Competence in Decision Rules (CDR) is a subtest of the Adult Decision-Making Competence battery [20,47] and assesses the ability to make financial decisions based on specific rules or criteria, such as selecting a product that meets certain requirements. The CDR contains ten scenarios with increasing complexity during which participants need to indicate which of five televisions (the original version referred to a DVD-player) they would buy using different decision rules. For example, ‘Emma wants the television with the highest average rating. However, she also wants to make sure that she buys a television that obtained the best rating on sound quality’. All televisions are equally priced, but vary in their technical specifications (e.g. picture quality and brand reliability). To make correct decisions in this test, participants are required to analyze the specifications of each television and to balance all pros and cons of a specific television based on the given rule. A total score is calculated based on the total number of correctly answered scenarios (maximum score is 10).

The Temporal Discounting Task (TDT) consists of eighteen different hypothetical scenarios and is used to assess decisions with implications for the future, such as choosing between spending or saving money. A critical characteristic of these decisions is that the subjective value of an option decreases over time (i.e. temporal discounting (TD) [48]). During each scenario of the TDT participants have to indicate the lowest amount of money they would accept today or after a relatively short delay instead of receiving a relatively high amount of money later in time (e.g. ‘Which amount of money would you accept today instead of receiving €100 in one year?’). Six different time intervals are used (i.e. ‘today vs. one week’, ‘today vs. one month’, ‘today vs. one year’, ‘one week vs. one month’, ‘one week vs. one year’ and ‘one month vs. one year’), which are combined with an amount of money participants can receive after a delay (i.e. €100, €500 or €1000). All answers are converted to a percentage of the amount of money participants can receive after a relatively long delay. Subsequently, one average score is calculated based on all six time intervals. A high score is indicative for a decreased sensitivity to TD and, therefore, the ability to make decisions with implications for the future.

The Impulsive Buying Questionnaire (IBQ) is used to investigate the tendency to buy on impulse, which can be described as the irrepressible tendency to perform a sudden unplanned purchase [49]. The IBQ assesses the affective, cognitive and situational components of impulsive buying [50–52]. The affective component refers to emotions and feelings that lead to impulsive buying behavior; the cognitive component refers to thoughts of and the urge to buy on impulse. A situational component, i.e. the availability of time and money that are needed to execute an impulsive purchase, also appears to be involved [50]. The situational component is, however, still experimental and therefore not part of the total IBQ score. All questions (e.g. ‘When I go shopping, I buy things that I did not intend to purchase’ or ‘I always buy it if I really like it’) are rated on a four-point scale, which ranges from 1 (strongly disagree) to 4 (strongly agree). Sum scores are calculated for the affective, cognitive and situational component. Furthermore, a total score is calculated reflecting the overall tendency to buy on impulse. Scores are reversed so that high scores represent low levels of impulsive buying tendency.

A computerized version of the Iowa Gambling Task (IGT) [53,54] was developed to assess real-life decision-making, however, seems to assess primarily emotional decision-making in healthy individuals [55]. Emotional decision-making in the context of the IGT can be described as an unconscious urge or feeling based on previous experience and emotional stage which has an influence on decision-making [55,56]. Participants have to choose cards from four decks and each deck is associated with certain gains and losses. The disadvantageous decks A and B lead to relatively high gains but also to relatively high losses. Decks C and D, the advantageous decks, result in relatively low gains but also in relatively low losses. Participants are not explicitly informed about the rules of winning and losing. They therefore have to learn from trial and error which decisions are most advantageous. A total netscore, i.e. the number of times a participant chose the advantageous decks minus the number of times the participant chose the disadvantageous decks, over 100 trials is calculated. Furthermore, the netscore will be divided in five parts of 20 trials to assess the effect of feedback and risky decision-making [57].

The Financial Decision-Making on Intuition or Deliberation test (FDM-I/D) is a computer test based on previous published procedures [58,59] and is used to assess intuitive and deliberative decision-making. This test is based on the theory that individuals who were distracted before they had to make a complex decision made significantly better decisions compared to individuals who made their decisions consciously and deliberately [58–60]. During the FDM-I/D participants are presented with information about attributes of four options of a hypothetical product (i.e. a boat, an apartment, a car and a flight ticket) of which they have to choose the best option. The attributes are either positively or negatively formulated and the options per product are designed in such a way that one option is the best product with 75% positive attributes. For two options the positive and negative attributes are equally represented, and the last option has only 25% positive attributes. During two trials (i.e. boat and apartment) participants were asked to base their decision on their intuition; i.e. participants are distracted for 2.5 minutes before they have to make a decision by performing a Go-NoGo test immediately after the presentation of the attributes. In the other two trials (i.e. car and flight ticket) participants are instructed to make their decision after deliberatively thinking about the positive and negative attributes during a period of 2.5 minutes. Participants received 0 points if they choose the best option, -25 points if they choose one of the options with 50% positive attributes and -50 points if they choose the option with only 25% positive attributes. A total score is calculated for intuitive-based and deliberative-based decisions by adding the scores on the intuitive-based trials and deliberative-based trials, respectively.

#### Neuropsychological tests

A neuropsychological assessment was performed to assess a wide range of cognitive functions, such as memory, attention, executive functions, psychomotor speed and numeracy. Several standardized, reliable and valid neuropsychological tests were used to assess these cognitive functions. Verbal short-term memory was examined with the Digit Span forward of Wechsler Adult Intelligence Scale IV (WAIS-IV—Digit Span forward [61,62]) and with the immediate recall of the Dutch version of the Rey Auditory Verbal Learning Test (RAVLT immediate recall [63]). Verbal long-term memory was assessed with the RAVLT delayed recall [63]. Psychomotor speed was assessed using the Trail Making Test—Part A (TMT A [64]) and the Stroop Color-Word Test—Word Card (Stroop card 1 [65]). Selective attention was examined with the D2 Test of Attention [66,67] (target measures: D2 Concentration Performance and D2 Total Correct). The following tests were used to assess different aspects of executive functioning: response inhibition—Color-Word Card/Color Card of the Stroop Color-Word Test (Stroop card 3–2 [65]); cognitive flexibility—Trail Making Test—Part B divided by Trail Making test—Part A (TMT B/A [64]); Semantic alternating fluency test (i.e. alternating between names of places and clothes during 1 minute [68]) and Phonemic alternating fluency test (i.e. alternating between words starting with the letters K and O during 1 minute [68]); planning—Tower of London Test (TOL); working memory—WAIS-IV Digit Span backward and WAIS-IV Digit Span sorting [61,62]; divergent thinking—Semantic fluency test (categories ‘Animals’ and ‘Professions’ [69]; each during 1 minute) and Phonemic fluency test (categories words starting with a ‘D’, ‘A’ and ‘T’ [70]; 1 minute for each letter). Finally, mathematical reasoning and numeracy were assessed with the WAIS—IV Arithmetic [61,62].

#### Data analyses

Statistical Package for the Social Sciences 23 was used for data analyses.

To determine the effects of age on the performance on the FDM tasks, thirteen hierarchical multiple regressions were performed on sample 1 using total scores of the FDM tasks as dependent variables. In the first step (method: enter) demographic variables that might be of influence on FDM were included as independent variables, i.e. gender, years of education, employment status and annual year income. Age in years was included as an independent variable in the second step (method: enter). Results of step two will be presented, unless otherwise stated. Normality was violated regarding the TDT total score; therefore, an arcsine transformation for percentage data was used for the TDT (i.e. $2\text{arcsine}\sqrt{\frac{{\text{X}}_{\text{i}}}{100}}$ [71]). When significant effects of age on FDM were found and a summary or total score was used as dependent variable (i.e. FCAI, FDMI, IBQ and IGT) the hierarchical multiple regression analysis was repeated including the different subscales or components as dependent variables. A p-value ≤ .05 was considered statistical significant.

When significant effects of age on FDM were found, the internal and external validity of these results were examined. To determine the internal validity, bootstrap analyses were performed to control for overoptimism [72,73]. In the bootstrap resampling procedure, 1,000 random samples of the original sample size were drawn with replacement from sample 1. Bootstrap 95% Confidence Intervals (CI) were estimated for the regression coefficients (b-values) as a measure of accuracy of the original regression analyses. When the 95% CI of the bootstrap analyses did not include the value of zero, the internal validation was considered satisfactory. To examine the external validity of the significant results, the hierarchical multiple regression analyses, including bootstrap analyses for significant results, were repeated using sample 2. The b-values of the hierarchical multiple regression analyses and the bootstrap analyses in the second sample were compared to the 95% CI of the conducted bootstraps on sample 1 to evaluate the external validity of the found age effects [73,74]. When b-values of sample 2 were within the 95% CI of the bootstrap of sample 1, the external validity was considered to be sufficient.

To investigate a possible mediating effect of cognition on the relation between age and FDM, a four steps procedure by Baron and Kenny [75] was followed. Step one is described above, i.e., the investigation of the relation between age and FDM. For step two, thirteen additional hierarchical multiple regression analyses (method: enter) were performed to determine the relation between cognition and participants’ performance in FDM tests. Dependent variables were the total scores of each FDM test. Independent variables were the participants’ performances on the different standard neuropsychological measures. When standard neuropsychological measures are found to significantly relate to aspects of FDM, additional hierarchical multiple regression analyses (step three) will be performed to determine the effects of age on these measures of cognition. For both step two and three, we controlled for demographic variables (i.e. gender, years of education, employment status and annual year income) and a Bonferroni correction was applied to control for alpha-error growth in multiple testing (i.e. p < .0038 and p < .017 were considered significant for step two and three, respectively). When significant results were found in step two and three, additional hierarchical regression analyses (step four) were performed to investigate the effects of age and FDM while controlling for demographic variables and standard measures of cognition. A significant reduction in the relation between age and FDM, as determined by the Sobel test, is indicative of a mediating effect of cognition.

Finally, correlation analyses (Pearson) were used to determine the coherence between the FDM tests and to further explore the relation between FDM tests and standard neuropsychological measures. Bonferroni correction was applied to control for alpha-errors and p < .001 was considered significant.

## Results

### Age effects on FDM

No significant effects of age were found on the total scores of the FCAI-NL and FDMI and on the application of financial decision-making styles (FDS; Table 2). However, with regard to the FCAI-NL years of education (b = 0.79 [0.35, 1.23], p = .001) as well as gross annual year income (b = 1.4 [0.19, 2.60], p = .023) were found to be significant predictors. Number of years of education was also a significant predictor of the total score of the FDMI (b = 0.11 [0.04, 0.19], p = .003) and years of education and gross annual year income were both significant predictors of the ‘intuitive style’ of the FDS (b = -0.167 [-0.30, -0.03], p = .015 and b = -0.432 [-0.81, -0.06], p = .025, respectively). None of the other FDS styles (i.e. rational, dependent, avoidant and spontaneous) could be predicted by any of the control variables. Furthermore, no significant effects of age, or of any of the control variables, were found on the FDM-I/D, neither for deliberative decisions nor for intuitive decisions (Table 2).

**Table 2:** Results of multiple hierarchical regression analyses of sample 1 and sample 2.

	Sample 1					Bootstrap of sample 1			Sample 2			Bootstrap of sample 2		
	Control variables^a^		Age			Age			Age			Age		
	R^2^	p (F)	b	ΔR^2^	p (ΔF)	Bias	SE	95%CI	b	ΔR^2^	p (ΔF)	Bias	SE	95%CI
FCAI-NL total	.163	< .001*	-0.015	.000	.791	-	-	-	-	-	-	-	-	-
FDMI total	.091	.003*	-0.008	.005	.358	-	-	-	-	-	-	-	-	-
FDS rational	.022	.488	0.003	.000	.848	-	-	-	-	-	-	-	-	-
FDS intuitive	.080	.002*	0.021	.008	.237	-	-	-	-	-	-	-	-	-
FDS dependent	.028	.361	-0.018	.005	.354	-	-	-	-	-	-	-	-	-
FDS avoidant	.012	.747	0.019	.007	.310	-	-	-	-	-	-	-	-	-
FDS spontaneous	.035	.233	-0.019	.010	.196	-	-	-	-	-	-	-	-	-
CD-R total	.211	< .001*	-0.066	.151	< .001*	0.001	0.010	[-0.085, -0.044]*	-0.070	.150	< .001*	< 0.001	0.017	[-0.101, -0.035]*
TDT total	.048	.088	0.006	.058	.001*	< 0.001	0.002	[0.002, 0.010]*	-0.001	.001	.770	-	-	-
IBQ total	.272	< .001*	0.120	.034	.011*	0.002	0.042	[0.035, 0.209]*	0.169	.062	.006*	< 0.001	0.040	[0.041, 0.198]*
Affective component	.257	< .001*	0.086	.063	< .001*	0.001	0.023	[0.042, 0.132]*	0.085	.048	.016*	< 0.001	0.038	[0.012, 0.163]*
Cognitive component	.215	< .001*	0.030	.007	.246	-	-	-	-	-	-	-	-	-
Situational component	.061	.044	-0.011	.015	.119	-	-	-	-	-	-	-	-	-
IGT netscore total	.017	.572	-0.619	.071	< .001*	0.011	0.174	[-0.959, -0.280]*	-0.040	.000	.878	-	-	-
Netscore trial 01–20	.018	.566	-0.035	.005	.343	-	-	-	-	-	-	-	-	-
Netscore trial 21–40	.042	.129	-0.112	.027	.032*	0.002	0.054	[-0.213, 0.004]	-0.056	.005	.453	-	-	-
Netscore trial 41–60	.015	.656	-0.057	.007	.275	-	-	-	-	-	-	-	-	-
Netscore trial 61–80	.018	.565	-0.171	.067	.001*	-0.002	0.052	[-0.279, -0.067]*	-0.020	.001	.790	-	-	-
Netscore trial 81–100	.027	.336	-0.252	.120	< .001*	0.001	0.052	[-0.353, -0.146]*	0.018	.000	.816	-	-	-
I/D ‘intuition’	.036	.217	-0.146	.014	.132	-	-	-	-	-	-	-	-	-
FDM-I/D ‘deliberation’	.043	.126	-0.113	.009	.223	-	-	-	-	-	-	-	-	-

Note.

^a^ Control variables included were gender, education (in years), income and employment status; ΔR^2^ = R^2^ change (i.e. added variance when including age); p(ΔF) = p-value F change; CD-R = Competence in Decision Rules; FCAI-NL = Financial Competence Assessment Inventory—NL; FDMI = Financial Decision-Making Interview; FDM-I/D = Financial Decision Making on Intuition or Deliberation; FDM-I/D = Financial Decision Making on Intuition or Deliberation Test; FDS = Financial Decision Styles; IBQ = Impulsive Buying Questionnaire; IGT = Iowa Gambling Task; TDT = Temporal Discounting Task;

* significant with p < .05.

The number of years of education significantly predicted the CDR performance (b = 0.17 [0.98, 0.25], p = < .001). However, age was found to be a significant additional predictor of the performance on the CDR, explaining 15.1% of variance; a finding that is supported by both the internal and external validation analyses (Table 2). Negative effects of age were also found on the IGT. The total netscore of the IGT was significantly predicted by age, explaining 7.1% of variance. Specifically, the second, fourth and fifth trial of the IGT were significantly predicted by age (Table 2). No significant effects of age were found for the first and third trial of the IGT and the control variables did not predict the performance on the IGT. The internal validation analyses partly supported these results. However, the results could not be replicated in the external validation analyses (Table 2).

Gender and gross annual year income were found to explain 27.2% of variance of scores on the IBQ (b = -5.55 [-8.32, -2.78], p < .001 and b = 1.20 [0.15, 2.25], p = .026, respectively). Women had lower IBQ scores, indicating a stronger impulsive buying tendency, than men. However, age was also a significant predictor of the IBQ, explaining an additional 3.4% of variance. Further investigation of the three IBQ components indicates that the relation of age is particularly evident for the affective component. Again, gender explained a large proportion of the variance (i.e. 25.7%; b = -3.27 [-4.70, -1.85], p < .001) of the IBQ affective component, but age was an additional significant predictor of this component and explained 6.3% of the variance. Both the internal as well as external validation analyses support the effects of age on the IBQ (Table 2). The cognitive and situational components of the IBQ were not significantly influenced by age. Regarding the TDT, no relation was found with any of the control variables, but 5.8% of the variance was explained by age (Table 2). The internal validation analyses support this finding, but these age effects lack external validity since these results could not be replicated in sample 2 (Table 2).

### Mediating effect of cognition

After controlling for gender (β = -0.15, p = .018) and years of education (β = 0.15, p = .015), standard measures of cognition explained additional and significant variance of the performance on the CDR (ΔR^2^ = .40, ΔF(17, 142) = 8.77, p < .001). Numeracy (Arithmetic) was the strongest predictor of the performance on the CDR (β = 0.27, p < .001), followed by planning (TOL) and working memory (Digit Span backward) (β = 0.19, p = .003 and β = 0.16, p = .023, respectively). The remaining twelve regression models focused on the effects of cognition on each of the FDM tests did not reach statistical significance (.056 ≤ p ≤ .915).

For numeracy (WAIS-IV Arithmetic), planning (TOL) and working memory (WAIS-IV Digit Span backward) the relation with age was investigated. Age was significantly negatively related to planning (TOL; ΔR^2^ = .14, β = -0.49, p < .001) and numeracy (WAIS-IV Arithmetic; ΔR^2^ = .05, β = -0.30, p = .002), whereas no significant relation was found between age and working memory (WAIS-IV Digit Span backward) (β = -0.15, p = .120).

A mediation analysis was therefore performed for the relation between age and the CDR controlling for TOL and WAIS-IV Arithmetic. The results indicate that, after controlling for these measures of cognition, the strength of the relation between age and the CDR decreases but remains significant (β = -0.30, p < .001 after controlling for TOL and WAIS-IV Arithmetic compared to β = -0.50, p < .001 in the original analysis). According to the Sobel test, the decrease in the association between age and CDR can be explained by a mediating effect of both numeracy (WAIS-IV Arithmetic, p = .003) and planning (TOL, p = .012).

Related to these findings, only a few weak correlations between standard neuropsychological measures and FDM tasks were found (S1 Table). Correlation analyses performed to determine the coherence between different FDM tests showed also only weak to negligible correlations (S2 Table).

## Discussion

The objective of this explorative study was to determine the effects of normal aging on different aspects of FDM. Based on previously published procedures in the field of (medical) decision-making, a comprehensive test battery was developed reflecting eight aspects of FDM. It was expected that some more cognitive demanding aspects of FDM were negatively influenced by normal aging due to age-related cognitive decline [13,14,17,18], while other aspects remained relatively stable or even improved due to an age-related increase of knowledge and experience or an improved affective decision-making in older adults [15,28–30].

When looking at the different aspects of FDM, no effects of normal aging were found on relatively basic aspects of FDM, i.e. financial competence and (financial) decision-making capacity. These results suggest that the (mental) ability or capacity and knowledge to perform actions that are needed to execute financial transactions on a relatively basic level are relatively stable across the adult life span. Furthermore, no or only relatively weak associations were found between financial competence, (financial) decision-making capacity and measures of cognition, suggesting that financial competence and (financial) decision-making capacity are not related to performances on measures of cognition. However, it is also possible that an age-related improvement in affective processing and an increased knowledge and experience with regard to these relatively basic aspects of FDM compensate for an age-related decline in cognition [31,32]. This hypothesis cannot be evaluated on the basis of the present data and therefore needs further investigation. Interestingly, most studies that examined the capacity to make decisions within a medical context, e.g., about different treatment options, among patients with mental disorders [76,77], medical disorders [40,41] or elderly with and without cognitive impairments [39,42], suggest that a poorer decision-making capacity is related to cognitive dysfunctioning. None of the studies, however, examined the effects of normal aging on the capacity to make decisions by using a life-span perspective. Furthermore, so far, only two studies focused on decision making capacity in a financial context and found that patients with mild cognitive impairments or Alzheimer’s disease [78] and patients with intellectual disabilities [38] have more difficulties with financial decision-making capacity compared to a healthy comparison group. Also the FCAI, measuring financial competence, has been shown to be able to distinguish individuals with cognitive impairments from individuals without cognitive impairments [6]. These studies suggest that financial competence and (financial) decision-making capacity are sensitive to more severe cognitive dysfunctions and, therefore, might be impaired in patients with e.g. mild cognitive impairments or Alzheimer’s disease. In the present study, however, no evidence for effects of normal aging on these relatively basic aspects of FDM were found.

Also regarding the use of decision-making styles in FDM situations no significant effects of normal aging were found. To our knowledge, the present study is the first study investigating decision-making styles in the context of FDM. Previous research on more general decision-making styles indicated that the use of intuitive and avoidant styles decrease with advancing age [45]. The results of the present study, however, suggest that this does not apply to decisions in a financial situation. Also no significant effects of age were found for deliberative and intuitive FDM. It was expected that older adults would become better in making intuitive decisions since they rely more on the affective processes [15]. However, no evidence for this expectation was found. Since a recently published meta-analysis including 61 studies concluded that there is no evidence for previous suggestions that intuitive decisions in complex situations lead to better decisions than deliberative decisions [79], intuitive and deliberative decision-making were not directly compared in the present study.

With increasing age, the tendency to buy on impulse seems to decrease. Particularly the affective component of impulsive buying behavior appears to be sensitive to the effects of normal aging. Both the internal and external validation analyses support these findings. The results suggest that with advancing age individuals are less prone to feelings of temptation or immediate satisfaction when buying something, which is consistent with improved affective processes [15,28,29] and a better impulse control or emotion-regulation that accompanies aging [80]. Related to the improved impulse control with advancing age, it was found in sample 1 that with advancing age individuals are less sensitive to immediate rewards (TD); these results could, however, not be confirmed in sample 2. The results of previous studies on the effect of aging on TD are also mixed, with some studies demonstrating an increased sensitivity to TD with advancing age, while others show no effects of normal aging on TD (e.g. [48,81–84]). It has been hypothesized that income might have an effect on TD [83] and mediate the age effects. Also other financial resources, such as financial reserves, might be of influence on the association between normal aging and TD.

Inconsistent findings between sample 1 and 2 were also found for emotional decision-making measured with the IGT. An age-related decline was found in sample 1 for emotional decision-making. This was particularly evident for the last parts (i.e. the last forty trials) of the task, which are suggested to assess risky decision-making [57]. This age effect is in accordance with previous research [85–87]. However, the internal validation analyses only partly supported these findings and results could not be replicated by the external validation analysis, i.e. no age effects on the IGT were found in sample 2. A possible explanation for these discrepant findings might be that the two samples included in the present study differed with regard to number of years of education. Indeed, several studies showed that performance on the IGT might be affected by level of education [88,89].

Nevertheless, the present study did find strong evidence that the ability to apply rules during a FDM situation (i.e. CDR) decreases with advancing age. This result indicates that when (financial) decisions need to be made based on specific preferences or rules, older adults have more difficulties with applying these preferences or rules than younger individuals. Both the internal and external validation analyses support this finding. Previous research suggested that this decline in FDM can be attributed to a decrease in deliberative processes and executive functioning [13–15,21], in particular inhibition [90] and working memory [91]. Indeed, in the current study 40% of variance of the performance on the CDR was explained by numeracy, planning and working memory, indicating that the ability to apply rules is a complex and cognitively demanding aspect of FDM. Furthermore, numeracy and planning appear to mediate the effects of aging on the CDR, however, these cognitive functions cannot completely explain the relation between age and this aspect of FDM. Furthermore, no evidence was found for a compensation effect of affective decision-making or experience for the performance on the CDR. Taken together, these results indicate that besides an age-related cognitive decline, also other variables are related to age-related difficulties in (financial) decision-making based on preferences and rules.

In summary, normal aging appears to have a differential effect on various aspects of FDM. Regarding relatively basic aspects (i.e. financial competence, (financial) decision-making capacity, financial decision styles and the quality of intuitive and deliberative decisions) no evidence for effects of normal aging were found. On the other hand, an aspect of FDM that has been associated with affective processes (i.e. impulsive buying tendency) seems to improve with advancing age. Furthermore, a more complex aspect of FDM (i.e. the ability to apply rules) appears to deteriorate with advancing age. Whether or not normal aging has an effect on FDM with implications for the future and emotional decision-making remains inconclusive. The possible influence of third variables (i.e. personal finances and level of education) needs further investigation in order to provide more insight into the effects of normal aging on these aspects of FDM.

When exploring the associations between performances on neuropsychological tests and FDM tests only weak and non-significant associations were found (S1 Table). This indicates that standard neuropsychological tests cannot be used to evaluate one’s ability to make financial decisions, which emphasize the importance of the development of reliable FDM measurements. However, it must be noted that we might have failed to reveal existing effects (beta-errors) due to a conservative method to control for alpha-error growth in multiple testing (i.e. Bonferroni correction). Furthermore, the inclusion of a healthy sample without distinct cognitive dysfunctions may also account for the lack of coherence between cognition and aspects of FDM due to small inter-individual differences or ceiling effects within the sample. A sample of individuals with distinct cognitive impairment might show stronger relations between aspects of FDM and measures of cognition. Finally, when exploring the associations between the different FDM tests, it was found that all correlations were of a weak to negligible size (S2 Table). This shows that there is no or only little overlap between the tests which supports the assumption of the existence of multiple independent aspects of FDM.

A limitation of the current study is that the education level of our sample was slightly higher than the average education level in The Netherlands. This problem of representativeness of samples is, unfortunately, a common problem that is difficult to counter in scientific research with humans [92]. Second, the eight aspects of FDM might not reflect all FDM situations an individual may encounter in daily life. Furthermore, in spite of the theoretical classification of different FDM aspects and the weak correlations found between FDM tasks, it is possible that in some cases certain tasks relate to similar constructs or aspects of FDM. A third limitation of the present study is that the convergent validity could not be evaluated since there are hardly any additional FDM tests available. In addition, the ecological validity of the FDM tests was not examined so far by exploring the associations between the performances on the FDM tests and real-life FDM problems. Fourth, it must be noted that the effects of age on FDM might have been confounded by the fixed sequence of test administration and differences in the assessment procedure of sample 1 and 2 (S1 Procedure). However, by using the same sequence of FDM tests in sample 1 and 2 and providing participants the opportunity for as much breaks as needed the effects of the test administration order were likely kept on a minimum. Finally, the role of financial experience and knowledge was not taken into account sufficiently.

Nevertheless, by investigating multiple aspects of FDM, a better understanding of age-related differences in FDM was obtained. Normal aging influences some aspects of FDM, while other aspects appear to be unaffected by normal aging. Age-related decline in FDM, which is possibly related to normal age-related changes in the prefrontal networks [11,12], seems to be mediated by age-related changes in executive functions [13,14], particularly in planning and numeracy. However, also working memory was found to be predictor of FDM. Improved affective decision-making and an age-related increase of knowledge and experience in older individuals [15,28–30] might result in stable or improved FDM with advancing age. In future research the role of motivation during complex FDM situations, such as applying decision rules, needs further investigation since an older age has been associated with a lack of motivation for difficult or complex (decision-making) situations [93]. Furthermore, it would be interesting to further explore individual differences in FDM by focusing on the effects of gender, financial resources and level of education, since it is generally known that demographic characteristics have an effect on neuropsychological test performances [65,94,95] and might have a mediating or moderating influence on the effects of age on FDM. In addition, studying FDM in patient groups, such as patients with neurodegenerative disorders, attention deficit hyperactivity disorder or traumatic brain injury, is of utmost importance since it can be expected that these patients experience problems with FDM in daily life due to more severe impairments in cognition (e.g. [9,24,78,96–98]) than found in healthy (older) adults. Finally, it is relevant to relate the current FDM tests to everyday situations and problems in FDM to determine the ecological validity of the different FDM tests.

## Supporting information

S1 Procedure Sequence of tests in sample 1 (and 2). (DOCX)

S1 Table Correlations between FDM tests and standard neuropsychological measurements (Pearson). (DOCX)

S2 Table Correlations between FDM tests (Pearson). (DOCX)
