# Supplementary material for: The effects of normal aging on multiple aspects of financial decision-making
Source: PLoS One. 2017 Aug 9;12(8):e0182620. doi: 10.1371/journal.pone.0182620 (PMC5549929; doi:10.1371/journal.pone.0182620)
Supplement: S1 Procedure — (DOCX) [file pone.0182620.s001.docx]

**S1 Supporting Information – Sequence of tests in sample 1 (and 2).**

Questionnaires (e.g. Financial Decision Styles questionnaire* and Impulsive Buying Questionnaire*) were completed at home prior to assessment.

Start of assessment

- Short demographic characteristics questionnaire (e.g. age, gender, education)
- Mini-Mental State Examination (MMSE)
- Financial Competence Assessment Inventory (FCAI)*
- Rey Auditory Verbal Learning Test (RAVLT) – Immediate recall
- Tower of London (TOL)
- Trail Making Test (TMT)
- D2 Test of Attention (D2)
- Rey Auditory Verbal Learning Test (RAVLT) – Delayed recall and recognition

*Optional break***

- Financial Decision Making Instrument (FDMI)*
- Competence in Decision Rules (CDR)*
- Semantic fluency

*Mandatory break (15-20 min.)***

- Iowa Gambling Task (IGT)*
- Emotion Focused Financial Decision-Making (EF-FDM)*

*Optional break***

- Wechsler Adult Intelligence Scale IV – Arithmetic
- Phonemic fluency
- Wechsler Adult Intelligence Scale IV - Digit Span
- Temporal Discounting Task (TDT)*
- Semantic and Phonemic alternating fluency
- Stroop Color-Word Test (STROOP)

End of the assessment

* The Financial Decision-Making tasks were assessed in sample 2 in the same sequence as in sample 1.

** Besides the mandatory and optional breaks, participants could receive as many breaks as needed between the tests upon request.
