# Supplementary material for: The effects of normal aging on multiple aspects of financial decision-making
Source: PLoS One. 2017 Aug 9;12(8):e0182620. doi: 10.1371/journal.pone.0182620 (PMC5549929; doi:10.1371/journal.pone.0182620)
Supplement: S1 Table — (DOCX) [file pone.0182620.s002.docx]

**S1 Table. Correlations between FDM tests and standard neuropsychological measurements (Pearson).**

|  | | | | | | | | | | | | | FDS | | | | | | | | | | | FDM-I/D | | | | |
| --- | --- | --- | --- | --- | --- | --- | --- | --- | --- | --- | --- | --- | --- | --- | --- | --- | --- | --- | --- | --- | --- | --- | --- | --- | --- | --- | --- | --- |
|  | FCAI-NL | | FDMI | | CDR | | IBQ | | IGT | | TDT | | | Rational | | Intuitive | | Dependent | | Avoidant | | Spontaneous | | | Intuition | | Deliberation | |
| RAVLT immediate | .13 |  | .17 |  | .42 | * | -.31 | * | .17 |  | .04 |  | | -.06 |  | -.01 |  | .09 |  | -.03 |  | .18 |  | | .19 |  | -.02 |  |
| RAVLT delayed | .12 |  | .06 |  | .37 | * | -.31 | * | .19 |  | -.05 |  | | -.11 |  | .02 |  | .13 |  | .03 |  | .15 |  | | .18 |  | .02 |  |
| TMT-A | -.05 |  | .02 |  | -.29 | * | .22 |  | -.14 |  | .11 |  | | .16 |  | -.003 |  | -.20 |  | .002 |  | -.23 |  | | -.07 |  | .03 |  |
| TMT B/A | -.05 |  | -.15 |  | -.07 |  | .06 |  | -.13 |  | -.02 |  | | .01 |  | .07 |  | .08 |  | .05 |  | .01 |  | | -.01 |  | .06 |  |
| Stroop card 1 | -.01 |  | .01 |  | -.32 | * | .27 | * | -.12 |  | .10 |  | | .17 |  | .02 |  | -.16 |  | -.02 |  | -.18 |  | | -.08 |  | .14 |  |
| Stroop card 3/2 | -.05 |  | -.12 |  | -.40 | * | .14 |  | -.21 |  | -.01 |  | | .10 |  | .04 |  | -.08 |  | -.03 |  | -.12 |  | | -.12 |  | .05 |  |
| TOL | .15 |  | .19 |  | .50 | * | -.02 |  | .29 | * | .01 |  | | .15 |  | -.07 |  | .04 |  | -.06 |  | .06 |  | | .08 |  | .10 |  |
| D2 CP | .21 |  | .16 |  | .49 | * | -.13 |  | .22 |  | -.09 |  | | -.04 |  | -.07 |  | -.002 |  | -.10 |  | .10 |  | | .06 |  | -.10 |  |
| D2 Correct | .16 |  | .04 |  | .43 | * | -.10 |  | .21 |  | -.11 |  | | -.07 |  | -.04 |  | -.02 |  | -.11 |  | .11 |  | | .04 |  | -.08 |  |
| Digit Span forward | .15 |  | .18 |  | .36 | * | .03 |  | .22 |  | .003 |  | | .15 |  | -.10 |  | .02 |  | -.04 |  | -.02 |  | | .17 |  | -.05 |  |
| Digit Span backwards | .16 |  | .20 |  | .47 | * | .02 |  | .20 |  | .01 |  | | .08 |  | -.06 |  | -.01 |  | .03 |  | .04 |  | | .11 |  | .07 |  |
| Digit Span sorting | .13 |  | .22 |  | .47 | * | -.16 |  | .23 |  | -.06 |  | | -.01 |  | -.15 |  | .01 |  | -.12 |  | .15 |  | | .18 |  | .10 |  |
| Semantic fluency | .22 |  | .24 | * | .28 | * | -.07 |  | .15 |  | -.01 |  | | -.17 |  | -.07 |  | .11 |  | .02 |  | .11 |  | | .12 |  | -.17 |  |
| Phonemic fluency | .16 |  | .09 |  | .30 | * | -.06 |  | .16 |  | .003 |  | | .003 |  | -.08 |  | .06 |  | .02 |  | -.08 |  | | .15 |  | -.07 |  |
| Semantic Alternating | .13 |  | .18 |  | .31 | * | -.01 |  | .18 |  | .10 |  | | -.01 |  | -.21 |  | .03 |  | -.03 |  | -.03 |  | | .04 |  | -.04 |  |
| Phonemic Alternating | .17 |  | -.01 |  | .34 | * | -.11 |  | .22 |  | -.01 |  | | -.07 |  | .03 |  | .16 |  | .02 |  | .03 |  | | .12 |  | -.02 |  |
| WAIS-IV Arithmetic | .33 | * | .16 |  | .58 | * | .08 |  | .24 | * | .11 |  | | .13 |  | -.23 |  | -.09 |  | -.11 |  | -.01 |  | | .03 |  | .10 |  |

Note. FCAI-NL = Financial Competence Assessment Inventory – NL; FDMI = Financial Decision-Making Interview; CDR = Competence in Decision Rules; FDS = Financial Decision Styles; TDT = Temporal Discounting Task; IBQ = Impulsive Buying Questionnaire; IGT = Iowa Gambling Task; FDM-I/D = Financial Decision-Making on intuition or deliberation; RAVLT = Rey Auditory Verbal Learning Test; TMT = Trail Making Test; TOL = Tower of London; D2-CP = D2 Concentration Performance; WAIS-IV = Wechsler Adult Intelligence Scale IV; * p < 0.001**.**
