# Supplementary material for: The effects of normal aging on multiple aspects of financial decision-making
Source: PLoS One. 2017 Aug 9;12(8):e0182620. doi: 10.1371/journal.pone.0182620 (PMC5549929; doi:10.1371/journal.pone.0182620)
Supplement: S2 Table — (DOCX) [file pone.0182620.s003.docx]

**S2 Table. Correlations between FDM tests (Pearson).**

|  | | | | | | | | | | | | | FDM-I/D | |
| --- | --- | --- | --- | --- | --- | --- | --- | --- | --- | --- | --- | --- | --- | --- |
|  | FCAI-NL | | FDMI | | CDR | | IBQ | | IGT | | TDT | | Intuition | Deliberation |
| FDMI | .20 |  | - |  | - |  | - |  | - |  | - |  | - | - |
| CDR | .32 | * | .21 |  | - |  | - |  | - |  | - |  | - | - |
| IBQ | .07 |  | -.05 |  | -.10 |  | - |  | - |  | - |  | - | - |
| IGT | .10 |  | .19 |  | .34 | * | -.08 |  | - |  | - |  | - | - |
| TDT | .14 |  | .06 |  | -.06 |  | .35 | * | -.02 |  | - |  | - | - |
| FDM-I/D ‘Intuition’ | -.04 |  | .04 |  | .13 |  | -.07 |  | .10 |  | -.02 |  | - | - |
| FDM-I/D ‘Deliberation’ | -.09 |  | .14 |  | .15 |  | -.12 |  | .13 |  | .09 |  | .03 | - |
| FDS Rational | .24 |  | .02 |  | .13 |  | .39 | * | .03 |  | .20 |  | -.10 | .07 |
| FDS Intuitive | -.11 |  | -.19 |  | -.21 |  | -.28 | * | .04 |  | -.09 |  | -.08 | -.14 |
| FDS Dependent | -.11 |  | -.02 |  | -.03 |  | -.18 |  | .15 |  | -.01 |  | .15 | .08 |
| FDS Avoidant | -.13 |  | .02 |  | -.08 |  | -.15 |  | -.03 |  | .004 |  | -.12 | .08 |
| FDS Spontaneous | -.11 |  | .08 |  | .02 |  | -.55 | * | .06 |  | -.19 |  | -.003 | -.04 |

Note. FCAI-NL = Financial Competence Assessment Inventory – NL; FDMI = Financial Decision-Making Interview; CDR = Competence in Decision Rules; FDS = Financial Decision Styles; TDT = Temporal Discounting Task; IBQ = Impulsive Buying Questionnaire; IGT = Iowa Gambling Task; FDM-I/D = Financial Decision-Making on intuition or deliberation; * p < 0.001.
